# Intake of dietary folate and folic acid in Germany based on different scenarios for food fortification with folic acid

**DOI:** 10.1007/s00394-014-0781-1

**Published:** 2014-10-24

**Authors:** Yvonne Martiniak, Thorsten Heuer, Ingrid Hoffmann

**Affiliations:** Department of Nutritional Behaviour, Max Rubner-Institut, Federal Research Institute of Nutrition and Food, Haid-und-Neu-Str. 9, 76131 Karlsruhe, Germany

**Keywords:** Dietary folate intake, Folic acid, Fortification, Supplementation, German National Nutrition Survey

## Abstract

**Purpose:**

Besides the adverse health effects of a low folate intake, the risks of high intakes of folic acid have moved into the focus. The aim of this study was to investigate the potential range of folate and folic acid intake of the German population under consideration of different fortification scenarios.

**Methods:**

Food consumption data of 13,926 participants of the German National Nutrition Survey II (NVS II), collected with two 24-h recalls, were used to calculate the nutrient intake. The nutrient data are based on the German Nutrient Database (BLS), information from a market survey and analyses of multivitamin juices. The scenarios were modelled without, as well as with low and high fortification levels of folic acid.

**Results:**

The median intake of dietary folate equivalents ranged from 191 µg/d (men) and 168 µg/d (women) without fortification to 425 µg/d (men) and 334 µg/d (women) in the highest fortification scenario. Thus, 12.4–68.2 % (men) and 5.9–56.1 % (women) met the 300 µg/d recommended by the nutrition societies of Germany, Austria and Switzerland. In the highest fortification scenario, 1.9 % (men) and 0.8 % (women) exceeded the tolerable upper intake level (UL) of 1,000 µg/d folic acid given by the European Food Safety Authority. For supplement users, this proportion was 5.2 and 5.4 %.

**Conclusions:**

Only a high fortification of several foods leads to a marked increase of the proportion of population reaching the recommendation. Simultaneously, with a high fortification a higher proportion exceeds the UL, especially in combination with supplements.

## Introduction

Many studies show the health benefits of natural food folate (reduced methyl and formyl folates) and of folic acid from supplements and fortified foods (pteroylmonoglutamic acid). An adequate folate intake during the periconception period helps to prevent congenital malformations (including neural tube defects) and pregnancy complications [1–3]. Low levels of folate can lead to homocysteine accumulation [4] and impairment of DNA repair mechanisms [5]. Epidemiological studies have suggested an association between high intake of folate and lower risk of cardiovascular disease, some cancers and age-related cognitive decline [6–8]. However, the results of randomized controlled trials with folic acid did not support these findings in general [9–14].

In addition to the risks of a low folate intake, potential risks of high intakes of folic acid have moved into the focus of the discussion over the past years. New scientific evidence has emerged suggesting a possible link between high intake of folic acid and various types of cancer, particularly colorectal cancer [15–19]. Animal studies suggest that high folic acid intakes may have a dual effect: inhibiting the formation of neoplastic lesions in normal tissues and accelerating the malignant transformation of existing neoplasms [20]. Other postulated adverse effects of high folic acid intake include acceleration of cognitive decline during ageing (in combination with a vitamin B-12 deficiency) [21–23] and a reduction of the efficacy of antifolate drugs (used to treat cancer, epilepsy, rheumatic disease, psoriasis or malaria) [24–26]. A high intake of folic acid may result from supplements and/or from fortified foods.

To prevent neural tube defects, a mandatory fortification of flour with folic acid is practiced in over 50 countries, but has not been introduced in any European country [27]. In Germany, folic acid supplements are recommended during the periconceptional period [28] and folic acid is added to many foods, such as breakfast cereals, multivitamin juices (mixed fruit juice fortified with several vitamins) and other beverages, margarines, dairy products, sweets, convenience foods, and table salt. The amount of added folic acid varies widely between different products, brands, and storage time. Information on the consumption of fortified foods collected in nutrition surveys contains a degree of uncertainty, and actual folic acid contents in foods may differ from those presented in nutrient databases or on product labels.

Therefore, the objectives of this study were as follows:

1. to investigate the potential intake of dietary folate and folic acid of the German population based on the data of the German National Nutrition Survey II (NVS II) under consideration of different food fortification levels (no fortification with folic acid, low and high fortification level) and
2. to evaluate the percentage of the population meeting the recommended intake or exceeding the tolerable upper intake level (UL) of different food fortification levels.

The calculations were executed for the total population, for women of childbearing age (because of a more serious impact of a low intake) and for supplement users (because of a potentially higher risk of a too high intake of folic acid).

## Methods

### Nutrition survey

The intake of dietary folate and folic acid was calculated for 13,926 participants (6,257 men and 7,669 women) of the NVS II. The NVS II is a nationwide representative study conducted between November 2005 and January 2007 among German-speaking men and women between 14 and 80 years of age living in private households. The methods of the survey are described in detail elsewhere [29].

The nutrition data used for the present analysis were collected in two 24-h recalls. The participants were interviewed in detail about what they ate and drank on the day before, as well as about their supplement use during that day. The product names and quantity of the consumed supplements were recorded. For each participant, the interviews were carried out on 2 non-consecutive days with an average gap of about 16 days. The 24-h recalls were performed as computer-assisted telephone interviews using the programme EPIC-SOFT. This programme was developed within the study “European Prospective Investigation into Cancer and Nutrition” (EPIC) [30] and revised and adapted to the objectives of the NVS II [31]. The linkage of the consumed foods, beverages and supplements with a nutrient and a supplement database was conducted manually.

### Nutrient data

The calculation of the nutrient intake was done based on the German Nutrient Database (BLS) version 3.01 [32]. Further information was supplemented from a market survey on fortified foods and the analyses of folic acid in different multivitamin juices (mixed fruit juice fortified with several vitamins) both conducted at the Max Rubner-Institut in 2010. The calculation of folic acid intake from supplements was based on an internal Max Rubner-Institut supplement database which contains product information of supplements, e.g. product names and nutrient contents from the package, producers or internet, from 2006 to 2010.

Heterogeneous definitions of the different terms for “folate” require a carful description especially for the purpose of the presented study. The term “natural food folates” comprises only naturally occurring folates in foods while “folic acid” is understood as the synthetic form (pteroylmonoglutamic acid) used only in supplements and in fortified foods. The generic term “folate” refers to all compounds of folate.

Dietary folate equivalents (DFE) were estimated, because the bioavailability of synthetic folic acid consumed in a meal is 1.7 times higher than the bioavailability of natural food folate [28]. On the European market, the fortification of food with folic acid is regulated on the basis of Regulation No. 1925/2006 of the European Parliament and of the Council (amended in Regulation No. 1170/2009) [33, 34]. The declaration of folic acid on the label of fortified foods encompasses natural food folate plus added folic acid [34]. Therefore, the difference between the label value and the natural folate content (i.e. the folate content as given for a comparable non-fortified food by the BLS) was considered with a factor of 1.7. Afterwards, the amount of natural food folate was added again (label value = natural food folate + folic acid; DFE = natural food folate + 1.7 × folic acid).

### Dietary reference values

The DFE intake was compared to the recommendation of 300-µg DFE per day given in Germany, Austria and Switzerland [28] and in the Scandinavian countries [35] as well as to the lower recommendation of 200 µg/d given in Great Britain and the European Union [36, 37] and to the higher recommendation of 400 µg/d given in USA [38]. The intake of folic acid from fortified foods and supplements was compared to the UL of 1,000 µg/d by the European Food Safety Authority (EFSA) [39].

### Calculation of scenarios

Because of limited accuracy of the consumption of fortified foods and the wide range of fortification levels, four scenarios with different levels of folic acid fortification were calculated to estimate potential intake levels. For the calculation of the scenarios, the added amount of folic acid to foods were varied for all foods that are potentially fortified with folic acid, independent of information about fortification or brand names which may have been recorded in the 24-h recalls.

The following 4 scenarios were calculated with different levels of fortification:

#### *Scenario* 1 (*no fortification with folic acid*)

It was assumed that all participants consume no fortified foods, so that scenario 1 exclusively shows the intake of natural folate. All fortified foods which were named in the 24-h recalls were considered as non-fortified foods. In scenario 1a, the use of supplements was not considered, whereas in scenario 1b folic acid intake from supplements named in the 24-h recalls was considered.

#### *Scenario* 2 *and* 3 (*low and high fortification with folic acid of certain food groups*)

It was assumed that all participants consuming lemonades (soda), breakfast cereals, margarines, packet soups, cocoa powder and cocoa drinks, certain dairy products and certain sweets had consumed these foods with the lowest (scenario 2) or highest (scenario 3) fortification level among these food groups observed during a market survey (Table 1), regardless of whether the participants had declared the food item as “fortified”, “not fortified” or “fortification unknown” during the interview. The fortification level of multivitamin juices (mixed fruit juice fortified with several vitamins) was based on the analyses of the Max Rubner-Institut [40]. Scenario 3 included the maximum folic acid content directly after production and scenario 2 the minimum content after storage of 12 months (Table 1). The fortification level of other multivitamin and mixed fruit beverages diluted with water was calculated with a proportion of 50 % multivitamin juice.

**Table 1:** Amount of added folic acid which is used in the scenarios 2, 3 and 4

Food group	Scenario 2 (µg/100 g)	Scenario 3 (µg/100 g)	Scenario 4 (µg/100 g)
Multivitamin/mixed fruit juices^a^	71	245	245
Multivitamin/mixed fruit beverages (water diluted)^b^	36	123	123
Lemonade (soda)	30	30	30
Breakfast cereals	93	340	340
Muesli	6	76	76
Cereal bar	68	168	168
Cocoa powder	194	286	286
Milk (with fruit preparation/flavour)	9	25	25
Buttermilk (with fruit preparation/flavour)	30	30	30
Whey (with fruit preparation/flavour)	26	43	43
Yoghurt drink (with fruit preparation/flavour)	82	82	82
Margarine	100	1,000	1,000
Packet soup	18	25	25
Candies	300	800	800
Ice cream (sorbet)	47	47	47
Gummy candies	100	400	400
Dextrose	200	400	400
Diet drink	13	26	26
Diet powder	108	487	487
Protein powder	108	239	239
Energy powder	360	600	600
Protein drink	33	33	33
Table salt	0	0	10,000

^a^Analyses of folic acid conducted by the Max Rubner-Institut

^b^Values were calculated with a proportion of 50 % multivitamin juice

#### *Scenario* 4 (*high fortification with folic acid for certain food groups plus table salt fortified with folic acid*)

Scenario 4 is based on scenario 3. In addition, table salt used in the household was subjected to fortification with folic acid. Depending on the salt content (BLS) of the declared food items, a respective amount of folic acid (100 µg folic acid per gram salt) was calculated and added to all salted food items declared as homemade (preparation type: “homemade” in EPIC-SOFT).

### Statistical analysis

The DFE intake of a person was calculated as the mean of the two 24-h recalls each participant completed. Based on these data, the DFE intakes as well as the intakes of folic acid in the different groups are presented as median, 95 % confidence interval of median and 95th percentile. Differences in the frequency distribution of folic acid supplement use between men and women as well as age classes were tested by the chi-square test. The Jonckheere–Terpstra test was used to assess trends across age classes for supplement use. The nutrient intake data were weighted with regard to the data of the German official statistics (microcensus 2006, Federal Statistical Office) for sex, age, region, education, employment and household size. All statistical analyses were performed with SAS (version 9.2, SAS Institute Inc., Cary, North Carolina, USA). Boxplots were generated with SPSS (version 20.0, IBM Corporation, Armonk, New York, USA).

## Results

### Total population

**Fig. 1:**
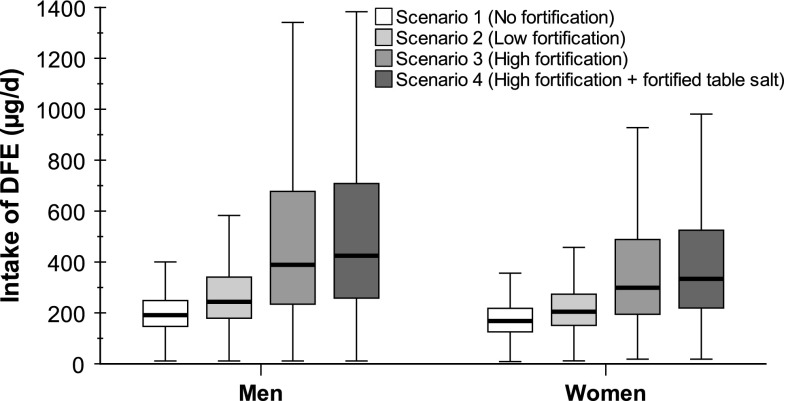
Intake of dietary folate equivalents (DFE) in the German population (*n* = 13,926) by gender for different fortification scenarios (excluding supplements). Boxplots show median, upper and lower quartiles and minimum and maximum data values excluding extreme values and outliers

The median daily intake of DFE (excluding supplements) ranged from 191 µg (scenario 1) to 425 µg (scenario 4) for men and from 168 to 334 µg for women (Fig. 1; Table 2). Without folic acid fortification (scenario 1), 12.4 % of the men and 5.9 % of the women met the recommended daily intake of 300-µg DFE of the German-speaking countries and the Scandinavian countries [28, 35]. In the scenario with the highest folic acid fortification level (scenario 4), 68.2 % of the men and 56.1 % of the women met this recommendation. The median intake for women also did not meet the lower Great Britain- and pan-European-recommendation of 200 µg/d [36, 37] with natural food folate in scenario 1, whereas men almost reached the recommendation of 200 µg/d. With high fortification (scenario 4), 86.0 % of the men and 73.3 % of the women met the recommended level of 200 µg/d. The higher recommendation of 400 µg/d in the USA [38] can hardly be reached without fortification. At the same time in the scenario with the highest fortification level (scenario 4), 1.9 % of the men and 0.8 % of the women exceeded the UL of 1,000 µg folic acid per day [39] (Table 2). In scenario 4, the 95th percentile of folic acid intake from fortified foods was 695 µg/d for men and 467 µg/d for women (Table 2).

**Table 2:** Intake of DFE and folic acid as well as the percentage of the German population meeting the recommended intake (200-µg, 300-µg and 400-µg DFE per day) and the percentage of the German population exceeding the UL (1,000 µg folic acid per day)

Scenarios	DFE^a^					Folic acid		
	Median (95 % CI)	P95^b^	≥200	≥300	≥400	Median (95 % CI)	P95^b^	>1,000
	(µg/d)	(µg/d)	µg/d^b^ (%)	µg/d^b^ (%)	µg/d^b^ (%)	(µg/d)	(µg/d)	µg/d^b^ (%)
Total population, men (*n* = 6,257)^c^								
1. No fortification	191 (189, 193)	366	45.7	12.4	3.1	0 (0, 0)	0	0.0
2. Low fortification^d^	244 (241, 247)	608	67.2	33.3	16.2	17 (16, 18)	224	0.0
3. High fortification^d^	389 (380, 400)	1,382	81.7	62.8	48.9	100 (95, 105)	674	1.8
4. High fortification^d^ + fortified table salt	425 (414, 434)	1,419	86.0	68.2	53.0	123 (116, 128)	695	1.9
Total population, women (*n* = 7,669)^c^								
1. No fortification	168 (166, 170)	310	32.8	5.9	1.3	0 (0, 0)	0	0.0
2. Low fortification^d^	205 (202, 207)	460	51.9	19.2	7.7	10 (9, 11)	142	0.0
3. High fortification^d^	299 (292, 306)	962	73.9	49.8	34.3	66 (63, 70)	446	0.8
4. High fortification^d^ + fortified table salt	334 (326, 341)	997	79.3	56.1	39.0	86 (82, 90)	467	0.8
Women of childbearing age (*n* = 3,720)								
1. (a) No fortification	159 (156, 164)	299	28.9	4.8	0.9	0 (0, 0)	0	0.0
1. (b) No fortification + supplements	165 (160, 171)	462	33.6	11.1	6.0	0 (0, 0)	100	0.4
2. Low fortification^d^	207 (200, 213)	512	52.6	22.0	9.8	15 (13, 18)	182	0.0
3. High fortification^d^	311 (296, 333)	1,106	74.6	52.9	36.8	78 (68, 87)	525	1.3
4. High fortification^d^ + fortified table salt	348 (332, 363)	1,142	80.0	58.6	41.8	100 (91, 109)	541	1.3
Folic acid supplement users, men (*n* = 442)								
1. (a) No fortification	207 (200, 211)	363	52.3	14.3	3.6	0 (0, 0)	0	0.0
1. (b) No fortification + supplements	488 (480, 500)	1,154	99.3	91.2	68.8	150 (150, 200)	500	1.6
2. Low fortification^d^ + supplements	554 (540, 565)	1,303	99.6	95.0	79.0	202 (200, 208)	627	1.6
3. High fortification^d^ + supplements	749 (722, 773)	1,948	99.8	97.3	89.1	314 (300, 325)	962	4.8
4. High fortification^d^ + fortified table salt + supplements	786 (746, 812)	2,006	99.8	98.2	91.4	332 (320, 351)	1,022	5.2
Folic acid supplement users, women (*n* = 745)								
1. (a) No fortification	187 (177, 195)	336	42.6	8.7	2.3	0 (0, 0)	0	0.0
1. (b) No fortification + supplements	500 (472, 527)	1,266	98.5	87.4	65.8	200 (200, 200)	600	2.7
2. Low fortification^d^ + supplements	545 (511, 577)	1,323	99.1	93.0	73.4	204 (200, 215)	625	2.7
3. High fortification^d^ + supplements	663 (627, 714)	1,914	99.2	95.4	83.9	283 (257, 300)	1,000	5.0
4. High fortification^d^ + fortified table salt + supplements	697 (658, 738)	1,933	99.2	96.1	87.7	299 (275, 322)	1,020	5.4

*DFE* dietary folate equivalents, *UL* tolerable upper intake level, *CI* confidence interval, *P95* 95th percentile

^a^To calculate DFE, synthetic folic acid has been considered with a factor of 1.7

^b^Data represents short-term consumption and therefore the proportions of adults with DFE/folic acid intake above the recommended intakes and the UL may be an overestimation

^c^Supplement use not considered

^d^Foods considered for fortification: multivitamin juices, mixed fruit beverages, lemonades (soda), breakfast cereals, margarines, packet soups, cocoa powder and cocoa drinks, certain dairy products, and certain sweets

### Women of childbearing age (15–45 years)

For women of childbearing age the median daily intake of DFE (excluding supplements) ranged from 159 µg (scenario 1a) to 348 µg (scenario 4) (Table 2). Without fortification with folic acid (scenario 1a), 4.8 % of the women between 15 and 45 years met the recommended daily intake of 300-µg DFE [28]. This proportion increased to 11.1 % when considering the use of supplements (scenario 1b). Without supplements and with a low fortification level (scenario 2), the percentage of women of childbearing age achieving the recommended intake of 300-µg DFE per day was higher (22 %), while no woman showed an intake of folic acid exceeding the UL. Under the assumption of a high fortification level (scenario 4), about 50 % met the recommended intake level and 1.3 % of the women of childbearing age exceeded the UL (Table 2).

In Germany, women of childbearing age are recommended to supplement 400 µg of folic acid per day in order to reduce their risk of a pregnancy affected by spina bifida or other neural tube defects. In this study, about 8 % of the women of childbearing age took supplements containing folic acid, but only 2 % at doses of at least 400 µg/d.

### Users of supplements containing folic acid

In Germany, more women (10 %) than men (7 %) used supplements containing folic acid (*P* < 0.001). The proportion of supplement users differed between age classes (*P* < 0.001, resp. for men and women) and increased across the age classes (*P* < 0.001, resp. for men and women) (Table 3).

**Table 3:** Use of supplements containing folic acid (%) by age classes

	Men (*n* = 6,257)	Women (*n* = 7,669)	*P* value*
Age**, *y*			<0.001
14–18	2.7	3.3	
19–24	5.0	8.2	
25–34	6.4	10.4	
35–50	7.6	8.6	
51–64	7.5	11.5	
65–80	9.1	11.9	
All	7.1	9.8	<0.001

* Significance of differences between men and women assessed by chi-square test

** Trends across age classes assessed by Jonckheere–Terpstra test were significant, *P* for trend <0.001

In the group of folic acid supplement users, the median daily intake of natural food folate was 207 µg for men and 187 µg for women (scenario 1a). Thus, the supplement users have a higher intake of natural food folate compared to the total population (Table 2). The median daily DFE intake including supplements ranged from 488 µg (scenario 1b) to 786 µg (scenario 4) for men and from 500 µg (scenario 1b) to 697 µg (scenario 4) for women (Table 2). Just with supplements (not considering fortified foods), the recommended daily intake of 300 µg [28] was achieved by 91.2 % of the men and 87.4 % of the women within this group. The median intake of supplemented folic acid was 150 µg/d for men and 200 µg/d for women, whereas the 95th percentile was 500 µg/d for men and 600 µg/d for women. In the scenario with the highest fortification level (scenario 4), 98.2 % of the men and 96.1 % of the women achieved the recommended 300 µg/d. At the same time, 5.2 % of the men and 5.4 % of the women exceeded the UL of 1,000-µg folic acid per day (Table 2).

## Discussion

The present study describes several scenarios of folic acid fortification based on the representative data of the NVS II. The results show a low intake of natural food folate (scenario 1) in Germany with respect to the recommended 300-µg DFE per day [28]. Without fortification, only 12.4 % (men) and 5.9 % (women) met this recommendation. A low folate intake has also been shown in previous studies in Germany [41, 42] and for most countries in Europe [43–45]. In Germany, major food sources for DFE are bread, vegetables, fruits, beverages as well as milk and dairy products [46].

In 2013, the nutrition societies of Germany, Austria and Switzerland lowered the recommendation for folate from 400 to 300 µg DFE per day for adolescents and adults (nonpregnant women) [28], now corresponding with the recommendation in the Scandinavian countries [35]. Even lower amounts (200 µg/d) are recommended in Great Britain and by the European Commission [36, 37], while in the USA the recommendation of the Institute of Medicine lies highest with 400 µg/d [38]. The current German recommendation of 300-µg DFE per day includes a safety margin and not meeting this intake level can therefore not be judged as an inadequate folate intake [28]. Taking this into account, different cut-off points based on different international recommendations are presented in this work. With respect to the lowest recommended intake of 200 µg/d (Great Britain and European Union) [36, 37], the folate intake in Germany is less critical.

The intake of DFE among German adolescents and adults can be increased by the consumption of fortified foods or of large amounts of one fortified food item (e.g. multivitamin juices).

Different fortification scenarios (partly in combination with supplements) reveal a wide range of potential folic acid intake levels. The consumption of foods with a low fortification level does not lead to a substantial increase of the proportion of the population meeting the recommendation of the German-speaking countries. However, with a high fortification level, almost 70 % of the men and about 55 % of the women met the recommended 300 µg/d, whereas about 80 % met the level of 200 µg/d. But this also results in a higher proportion of persons exceeding the UL (1.9 % of the men and 0.8 % of the women). The UL is an estimation of the maximum level of chronic daily intake, which carries no considerable risk of adverse health effects, whereas occasionally exceeding the UL does not seem to be a severe health threat [39].

The Scientific Committee on Food and the EFSA have set the UL for folic acid at 1,000 µg/d [39] because higher doses of folic acid could delay the diagnosis of vitamin B-12 deficiency by masking the anaemia of vitamin B-12 deficiency, which can lead to irreversible neurological damage. With respect to a potential tumour progression, the current UL of 1,000-µg folic acid per day may be too high, but the EFSA Scientific Cooperation Working Group concluded that there is no sufficient data to allow a full quantitative risk assessment of folic acid and cancer [27]. The possible harm of high folic acid intake depends most likely on additional factors such as age, individual supply status (e.g. vitamin B-12 status) and genetic polymorphisms [15, 21, 47–50]. With a lower UL, the percentage of persons exceeding the UL would be higher than presented in this study.

Particularly for women of childbearing age, a deficient intake can have serious consequences (neural tube defects or other prenatal malformations) but the intake of natural food folate within this group was even lower than within the total group of women. Without fortification with folic acid, only 4.8 % of the women between 15 and 45 years met the recommended daily intake of 300 µg DFE (scenario 1a). With a high fortification level of foods, 58.6 % met this recommendation (scenario 4). In Germany, it is recommended that women of childbearing age take an additional amount of 400 µg folic acid via supplements [28]. However, the proportion of woman meeting this recommendation was very low. This underlines that efforts have to be continued for more education about a diet rich in folate (such as green vegetables, fruits and wholegrain-products), fortified foods and a well-timed use of folic acid supplements as important prevention measures to reduce the risk of a pregnancy affected by neural tube defects.

The proportion of adults with a folic acid intake above the UL increases if folic acid supplements are taken (about 5 % among supplement users). The proportion of persons with folic acid supplement use is higher in the upper than in the lower age groups. A high intake of folic acid within the upper age groups may lead to critical situations due to a higher probability of occurrence of vitamin B-12 deficiency and/or undiagnosed preneoplastic lesions whose progression might be promoted by folic acid. Taking a closer look at the supplement users reveals a higher intake of natural food folate compared to the total population. These results suggest that particularly health-conscious people take folic acid supplements, who may not really need them, as well as elderly persons, who may have a higher health risk with high intakes of folic acid.

Because of the current fortification practice, i.e. products changing frequently and an amount of added folic acid that varies widely, the calculation of folic acid intake includes an uncertainty for scientific investigations and for consumers. Corresponding products from different brands are offered without fortification and with fortification at different levels. In addition, the exact content of folic acid in fortified foods is not known. In the European Union, the producers only have to declare the sum of natural folate and the added amount of folic acid on the label [34]. Furthermore, due to the instability of folic acid, variability in the declared content of ±30 % is accepted in Germany [51]. In some cases, including fruit juices, the tolerable deviation to the declared content may be more than +50 %. In other countries, a wide variability regarding the declared value is also reported for beverages, bread and breakfast cereals with −64 to +198 % [52] and −2 to +220 % [53].

In the present calculation, the variability of the folic acid content in multivitamin drinks (mixed fruit juices fortified with several vitamins, 100 % juice or water diluted) was considered (Table 1). Analyses of folic acid in nine multivitamin juices at the Max Rubner-Institut confirm the variability of the folic acid content depending on brand and storage time [40]. The consumption of several fortified foods or large amounts of one fortified food item (beverages especially can be consumed quickly in large amounts) may lead to a high intake of folic acid. At the individual level, the UL can easily be reached, e.g. by one glass of multivitamin juice (250 mL) plus 60 g breakfast cereal plus 250 g yoghurt drink (all with the highest fortification level of the corresponding food group). With multivitamin juice consumed right after production, the UL can be reached by consuming only 600 mL multivitamin juice. Considering the high contents of folic acid in freshly bottled multivitamin juices, the results support the discussion regarding the need for a restriction of folic acid fortification in selected foods such as beverages.

The estimates in the current study are subjected to several limitations. A limitation to dietary assessment methods is underreporting [54]. With the 24-h recalls used in NVS II, the energy intake is underestimated for 23 % of the study participants. Underreporting was calculated by the quotient (=cut-off level) of energy intake and resting metabolic rate considering sex, age, body height and body weight [55–57]. An exclusion of these so-called under-reporters and further of adolescents and of persons with incomplete data with respect to physical activity, body height and weight in further calculations of the Max Rubner-Institut (data not shown) leads to a higher median DFE intake of 20 µg/d for the remaining group.

In general, data from 24-h recalls reflect short-term food consumption. A statistical method for estimating usual food consumption distribution was not applied to the calculations in this study. Methods such as the National Cancer Institute Method (NCI) [58] or the Multiple Source Method (MSM) [59] correct the within-person variation of short-term measured data to reach a narrower intake distribution. Even if an overestimation of the presented proportions of adults with DFE intake above the recommended intakes cannot be excluded, exceeding the UL remains a relevant subject.

Another limitation is that in the present study only the dietary intake of natural food folate and folic acid could be considered, but not the folate status (e.g. folate concentrations in red blood cells). The latter depends on multiple factors as intake of folate and other factors, e.g. interactions with other nutrients (e.g. vitamin C) and genetic polymorphisms [60] and may lead to partly different results.

The scenarios 2, 3 and 4 are based on the assumption that all participants consuming multivitamin juices, mixed fruit beverages, lemonades (soda), breakfast cereals, margarines, packet soups, cocoa powder and cocoa drinks, certain dairy products, and certain sweets do this exclusively in a fortified form. It is hypothesized that the intake of folic acid in these scenarios reaches the theoretically possible maximum. However, there are some fortified foods, which could not be considered in the analysis, e.g. ready-to-bake bread mixes and convenience products. The higher stability of folic acid compared with natural food folate during cooking could not be included in the BLS calculation algorithm (scenario 4). Furthermore, adding salt at the table could not be considered in scenario 4, but only 5.5 % of the participants of the NVS II declared using table salt fortified with folic acid.

## Conclusion

In order to meet the recommendation of Germany, Austria and Switzerland, a substantial increase of the intake of DFE is necessary. This may be reached by a higher consumption of foods fortified with high levels of folic acid. Consequently, women of childbearing age with a low intake of folate benefit from foods fortified with folic acid and/or the use of supplements containing folic acid, especially if they become pregnant. On the other hand, more people with a high consumption of fortified foods and even more in combination with supplement use can reach intake levels of folic acid above the UL. The challenge is to increase the dietary intake of natural food folate or folic acid for women of childbearing age and at the same time lowering (or not increasing) the proportion of adults exceeding the UL.
